# Civic Engagement in Socially Excluded Young Adults Promotes Well-Being: The Mediation of Self-Efficacy, Meaning in Life, and Identity Exploration

**DOI:** 10.3390/ijerph19169862

**Published:** 2022-08-10

**Authors:** Irit Birger Sagiv, Limor Goldner, Yifat Carmel

**Affiliations:** 1 School of Creative Arts Therapies, Faculty of Welfare and Health Sciences, The Emili Sagol CATs Research Center, University of Haifa, Haifa 3498838, Israel; 2 Department of Educational Counseling, Faculty of Education, Beit Berl College, Kfar Saba 4490500, Israel

**Keywords:** civic engagement, well-being, hope, disadvantage, emerging adults, identity exploration

## Abstract

Civic engagement is thought to contribute to well-being among young adults. However, less is known about the ways in which civic engagement promotes well-being in general and in particular in socially excluded populations. This study investigated whether civic engagement contributes to life satisfaction and hope in a sample of 127 socially excluded young Israeli women who participated in social activism programs for a period of eight months. A mediation model incorporating self-efficacy, meaning in life, and identity exploration was used to examine the contribution of positive attitudes toward civic engagement, civic engagement skills, and political awareness to the participants’ life satisfaction and hope. Indirect effects were found between positive attitudes toward civic engagement, civic engagement skills, and political awareness and the participants’ life satisfaction and hope via self-efficacy. Positive attitudes toward civic engagement and political awareness also predicted the participants’ life satisfaction via meaning in life. A positive direct effect was found between political awareness and hope. However, contrary to the hypothesis, a negative direct effect was found between positive attitudes toward civic engagement and life satisfaction. Civic engagement skills and political awareness also predicted identity exploration. These findings underscore the need for clinicians to be aware of the potential benefits of civic engagement for the well-being of socially excluded populations.

## 1. Introduction

Social exclusion is defined as a state or process involving the denial of resources, rights, goods or services, and the inability to fully participate in social, economic, and political life [1,2]. This disturbing phenomenon is associated with a lack of finances and reduced well-being and happiness [3]. Despite constituting more than half the population, women experience significant social exclusion and inequality [4,5] which is reflected in higher poverty rates [6] and lower income levels [7], limited participation in the labor market [8], a marked absence from the public sphere, and less involvement in civic/political life than men [9]. No less concerning is the subjective experience of disadvantaged individuals, who tend to feel a decreased sense of belonging, self-esteem, control [10], and meaning in life [11], as well as higher levels of mental distress [12], and stress [13].

These negative indices are even more prominent during modern day emerging adulthood [3], a period characterized by delayed adulthood that is devoted to intensive identity exploration [4,14,15]. However, socially excluded young adults from lower socioeconomic backgrounds face unique stressors, more discrimination, and higher poverty rates in their transition to adulthood [16,17]. In addition, the need to enter the labor market quickly and milestones such as marriage and parenting [17,18,19] tend to provide socially excluded populations with fewer opportunities for self-exploration; thus, potentially hampering their health and well-being [20,21].

Social workers, developmental psychologists, and healthcare professionals all endeavor to facilitate socially excluded young adults’ well-being [22]. Most interventions addressing these young adults focus on subsidies [23], improving labor conditions [24,25], providing better housing [25,26], scaffolding education and professional training [23], and promoting health [27].

Nevertheless, interventions in recent years have shifted away from this type of deficit model toward a resilience paradigm targeting well-being and positive development trajectories. This change in orientation is grounded in the growing recognition that individuals’ strengths and not only the absence of mental or physical illnesses make a decisive contribution to optimal functioning [28,29]. Similarly, prominent theories in the field of positive youth development (PYD; [30,31]), positive psychology [32], and sociopolitical anti-oppressed development theories [33] suggest exercising civic engagement (generally defined as individual and collective actions that aim to address social issues, care for others in their communities, and fight for social change [34,35,36]) as a beneficial strategy that can strengthen the youngsters’ well-being [37].

For instance, Positive Youth Developmental (PYD) theory highlights the fundamental role of community engagement in shaping youth’s well-being, as manifested in the five Cs: competence, confidence, connection, caring, and character. These all constitute the platform for experiencing the sixth C; namely, contribution [38]. According to positive psychology, engaging in prosocial behavior and community engagement can accelerate happiness, life satisfaction, and hope by enhancing pathway and agentic thinking [32]. Finally, the sociopolitical and anti-oppressed development frame posits that civic engagement can enable young people to fight for the structural changes that can improve and empower individuals and communities [39].

Nevertheless, most studies have concentrated on examining the associations between civic engagement and well-being based on cohort data collected from American students or older adults (e.g., [36,37,40]). Few have examined the ways in which civic engagement advances the well-being of young adults. Much less is known about these trajectories in socially excluded populations, such as young women from low socioeconomic backgrounds supported by the welfare system, who have fewer opportunities for high-quality civic engagement [33]. To contribute to the literature, the current study examined the potential links between civic engagement and well-being as reflected in the life satisfaction and hope among socially excluded young women, as well as the mechanisms through which civic engagement promotes women’s well-being and hope.

### 1.1. Associations between Civic Engagement and Well-Being and Hope

Studies have indicated that engaging in significant, intentional, and beneficial behaviors allows people to feel good about themselves, feel they matter, and feel satisfaction in having made a contribution [39,41]. Findings based on self-report questionnaires examining a social protest movement in Israel 2011 found that adolescents with stronger feelings of meaning and coherence, whose value systems supported enhancing civic engagement and efficacy, were more hopeful and healthier [42]. In the context of oppression and sociopolitical development, civic engagement may provide a self-liberating mechanism from social oppression and can lead to the development of a critical awareness of societal dynamics, power relationships, and social justice [43,44,45]. This suggests that positive emotions and the enjoyment associated with social activity can relieve an often draining, exhausting, alienating, and muted reality [36].

For instance, a series of studies on adults and older adults reported an association between volunteering and life satisfaction [46,47,48,49] and positive affect [49]. A meta-analysis of 40 articles based primarily on cohort studies indicated that volunteering had favorable effects on depression, life satisfaction, and well-being [37]. Likewise, a longitudinal study that followed 9471 American adolescents into their early adulthood showed that civic engagement during adolescence was positively associated with lower levels of depression and risk-taking [50].

### 1.2. Self-Efficacy, Meaning in Life, and Identity Exploration as Mechanisms of Change

The favorable outcomes of civic engagement can also emerge indirectly through several self-constructs [51]. Since civic engagement is considered a strategy to promote one’s character [52], several studies have explored the role played by meaning in life and self-efficacy in contributing to satisfaction in life and hope [15,53,54]. Given the intense preoccupation with identity exploration in emerging adulthood, the role of identity exploration may also enhance young adults’ satisfaction in life and hope [55].

### 1.3. Civic Engagement and Meaning in Life

Meaning in life is defined in various ways related to coherence in one’s life, goal-directedness or purposefulness, or the ontological significance of life from the individual’s point of view [56]. In eudaimonic theories of well-being that focus on personal growth and strengths beyond positive and pleasant emotions, meaning in life is considered a critical component of well-being [57,58]. Because there is no universal definition appropriate for all, individuals need to create and ground their sense of meaning by pursuing significant goals or developing a coherent life narrative [56]. Studies have suggested that a sense of meaning can be achieved by addressing values, needs, and purposes [59]. Civic engagement, which is characterized by caring for others and goals on the macro-level, can thus lead to the realization of personal potential [60,61,62], which in turn may stimulate well-being [36].

### 1.4. Civic Engagement and Self-Efficacy

Likewise, civic engagement can promote self-efficacy (i.e., an optimistic self-belief that one can perform complicated tasks or cope with adversity; [63]). Creating change in one’s community may increase feelings of empowerment and hence overall well-being [36,50]. Because it involves wrestling with challenges and unexpected situations in new surroundings, civic engagement requires initiation, creativity, and the acquisition of communication and leadership skills, which can promote a sense of self-competence and efficacy [64].

### 1.5. Civic Engagement and Identity Exploration

Participating in civic engagement can facilitate the process of identity exploration. Theories of identity development can be traced back to Erikson’s dichotomous ego developmental model that described adolescence as the stage of identity acquisition versus role confusion [65,66]. Marcia [67] enlarged this model into four identity statuses consisting of identity achievement (commitment following exploration and experimentation), foreclosure (premature commitment), moratorium (ongoing investigation or avoidance), and diffusion (identity confusion). More recent theories have underscored the crucial interaction between commitment and exploration [68,69] and between commitment, reconsideration, and in-depth exploration [68,70,71], the opportunities to explore and agency [72], and the resources delivered by the social environment [38,73] to encourage agentic individualization governed by active opportunity-seeking behavior, choice, and personal growth [74,75].

Interacting with people of different ages, social strata, and ethnicities that differ from those one encounters regularly generates questions about one’s sense of self. These kinds of interactions encourage the exploration of one’s assets, values and goals, range of opportunities, and career choices. It can also help form networks of connections before making identity commitments [76]. In this respect, civic engagement can serve as a springboard to gaining social capital and shaping one’s future orientation more coherently [77,78]. This contribution is more significant for socially excluded groups who have fewer opportunities to be actively involved in identity exploration [78,79].

Through its anti-oppression perspective, civic engagement can help socially excluded populations gain inner and outer awareness, knowledge, and recognition of the ways societal power dynamics and institutional barriers shape their marginalized intersectional identity [80]. The ability to critically unpack the socio-political hierarchy and power relations while examining the interactions between outer context and intersectional self-identity may provide socially excluded volunteers with a more vigorous, sophisticated, and definite sense of subjectivity [53,81,82]. It can also allow volunteers to move from passivity to action and from silencing and oppression to empowerment; thus, contributing to their life satisfaction and hope [83,84].

Empirically, cross-sectional data have shown that involvement in civic engagement activities during the college years is associated with choosing a service career after college, increased self-competence, leadership, and interpersonal skills [85], as well as a greater commitment to future civic [81,86] and/or political involvement [87]. Students’ frequency of civic engagement was shown to be related to higher levels of feelings of personal efficacy [88] and purpose in life [89].

Students’ civic engagement also helped account for their purpose in life in adulthood [90]. A greater sense of meaning in life was reported among volunteering adults and chronically ill adults than among people who did not engage in such activities [91,92]. Civic engagement was related to general self-efficacy and self-esteem, which also served as mediators between volunteering and subjective well-being in survey data from Australia [93]. Finally, semi-structured interviews revealed agentic identity development in young people transitioning out of care after engaging in civic engagement [15].

### 1.6. The Current Study

The current study implemented a mediation model to examine the trajectories leading to well-being and hope through civic engagement in a sample of socially excluded young women. We concentrated on three cognitive-behavioral and emotional civic engagement dimensions stemming from this activism. These included attitudes and values related to the importance of the civic engagement, the skills needed, such as interpersonal communication, leadership, and problem-solving capabilities, and political awareness which was composed of critical and social justice thinking [94,95]. We addressed two questions: (a) To what extent are women’s positive attitudes toward civic engagement, civic engagement skills, and political awareness associated with their satisfaction in life and hope after eight months of civic engagement involvement? (b) Do meaning in life, self-efficacy, and identity exploration mediate the relationships between positive attitudes toward civic engagement, civic skills, and political awareness and these women’s satisfaction in life and hope?

**Figure 1:**
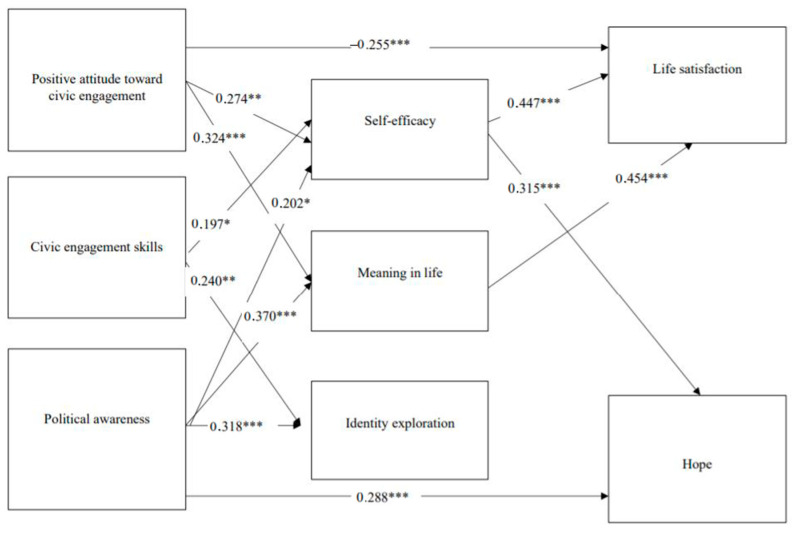
The results of the study model. * *p* < 0.05, ** *p* < 0.01, *** *p* < 0.001.

These considerations led to the following hypotheses: (a) High levels of positive attitudes toward civic engagement, civic skills, and political awareness should be associated with high levels of satisfaction in life and hope. (b) High levels of positive attitudes toward civic engagement, civic skills, and political awareness should be associated with high levels of women’s meaning in life, self-efficacy, and identity exploration. (c) Women’s levels of positive attitudes toward civic engagement, civic engagement skills, and political awareness should contribute directly to greater levels of women’s satisfaction in life and hope. In addition, it was hypothesized that women’s levels of meaning in life, self-efficacy, and identity exploration after eight months of involvement would mediate the associations between the dimensions of civic engagement (i.e., positive attitudes toward civic engagement, civic engagement skills, and political awareness) and satisfaction in life and hope (see Figure 1).

## 2. Method

### Participants and Procedure

A total of 127 socially excluded young Israeli women participated in a cross-sectional study. The data were collected as part of the “Young Women in the Lead—Social Activism in Young Women Adult Communities” project initiated by the Fund for Demonstration Projects of the Israel National Insurance Institute and the Gandyr Foundation. During this project, various organizations working with disadvantaged populations established 20 communities of socially excluded young adult women throughout the country. These women’s communities were characterized by multiple marginalization, including contextual and personal characteristics such as belonging to an ethnic minority group, residing in low-income peripheral towns, and a lack of family support. In some cases, the women had a history of child abuse and neglect or personal trauma, all of which were amplified as a result of their gender. In most cases, the women were recruited through welfare bureaus. In other cases, they were recruited by organizations that ran developmental programs they had taken part in as adolescents, or through other participants in the community. The goal was to encourage civic engagement as an innovative approach to promoting women’s well-being. The communities consisted of 5–10 participants who took part in weekly two-hour meetings in which designated coordinators introduced the women to various issues such as active citizenship skills and knowledge, political awareness, social equality, and gender.

The mean age of the participants was 21.16 (*SD* = 3.15). More than half were young women from the Arab sector of Israel (60.8%), and the rest were Jewish. Most of the participants were single (87.6%), 7.7% were married, and 4.7% were divorced. The majority of the participants (74.6%) lived with their parents, 16.2% lived in a rented apartment, 3.1% lived in housing projects, and 6.2% owned apartments. Fifty-three percent of the women had a part-time or full-time job. Thirty-one percent of the women defined themselves as secular, 30% traditional, 19% religious, and 20% as ultra-Orthodox. About a quarter (24.6%) of the women had up to nine years of schooling. In total, 16.9% had a high school education with or without a matriculation certificate, 17.7% graduated from technical school, 14.6% were students, and 26.2% had a college education. A series of ANOVA analyses did not reveal any differences between the participants as a function of their background variables or the study variables.

A series of self-report questionnaires were administered to assess the project’s effectiveness, relevance, and sustainability. The questionnaires presented in this study are part of a comprehensive questionnaire battery administered to the women. The questionnaires were translated from English into Hebrew or Arabic by the first two researchers and by two certified Arabic-speaking psychotherapists. Their translations were compared until a final version was acceptable to all. Disagreements were resolved by consensus. Since the study included high-risk women, we needed to shorten the questionnaires, eliminate intrusive items which could be interpreted as too revealing [96], simplify the language [97], and modify them to fit the specific setting of this study [98] to enhance the data quality and reduce missing data and inconsistent responses. In addition, to avoid confusion, the questionnaires were ranked on a five-point Likert type scale (1—*do not agree*; 5—*fully agree*). A pilot study was conducted to examine the reliability of the short version on 30 women.

After obtaining the approval of the Research Authority of the National Insurance Institute and the Gandyr Foundation, and the Ethics Committee of the Social Welfare and Health Faculty of the University of Haifa (approval number 18/310), all participants signed an informed consent form before completing the questionnaires. The questionnaires were filled out online on the Qualtrics platform in the communities or at a place of the women’s choosing. The participants filled out questionnaires on their attitudes toward civic engagement, civic skills, and political awareness and their levels of self-efficacy, meaning in life, identity exploration, life satisfaction, and hope. A trained research assistant provided face-to-face assistance or telephone assistance. Filling out the questionnaire took about half an hour.

## 3. Measures

### 3.1. Positive Attitudes toward Civic Engagement

Women’s level of positive attitudes toward civic engagement was assessed using four items from the duty dimension of the Civic Identity/Civic Engagement (CICE; [94]), which assesses individuals’ pro-civic attitudes and the desire and mindset to get involved with others to make positive contributions to society (e.g., how important is “helping to make the world a better place to live in?” adapted to “It is important for me to help to make the world a better place”). Studies indicate that civic participation can be predicted by civic duty [94]. The internal reliabilities (Cronbach’s alphas) in previous studies ranged from 0.73 to 0.91 [99,100]. The internal reliability (Cronbach’s Alpha) in the current study was 0.88.

### 3.2. Civic Skills

Three items from the interpersonal and problem-solving skills subscale and two items from the leadership subscale from the Civic Attitudes and Skills Questionnaire (CASQ; [95]) were used to assess the extent to which women could work collaboratively, communicate, and solve problems in a successful manner (“I can work cooperatively with a group of people”) and the extent of which they felt they could influence their surroundings effectively (“I have the ability to lead a group of people”). The internal reliabilities (Cronbach’s alphas) for both scales in the original study were 0.79. In addition, four items from the Civic Skills subscale from the Civic Identity/Civic Engagement inventory (CICE; [94]) were used to assess the extent to which the participants felt they had the ability and skills to be involved in social and democratic activities (e.g., (What is your ability to) sign an e-mail or written petition?). The internal reliability (Cronbach’s Alpha) in the original study was 0.91. The internal reliability (Cronbach’s Alpha) for the composite scale in the current study was 0.79.

### 3.3. Political Awareness

The women’s level of political awareness was assessed using two items measuring political and local awareness of national/local and political issues from the political awareness sub-scale on the Civic Attitudes and Skills Questionnaire (CASQ; [95]) (e.g., “I understand the issues facing my city/my community”). In addition, one item from the Evaluation of the Learning to Teach for Social Justice-Beliefs (LTSJ-B) Scale in an Australian Context [101] was added. This item assesses the extent to which the participants believed that political awareness was important (e.g., “People should be taught to think critically about the social policies of the state”). In addition, four items written specifically for this study were used to test the Israeli context and the context of the young adult women (e.g., “I understand the social problems facing groups in my environment including different SES groups, women’s groups, mothers, religious groups or different origins”). The Cronbach’s alpha for the CASQ (2002) ranged from 0.69 to 0.88 [95]. The Cronbach’s alpha for the LTSJ-B was 0.70 [101]. The internal reliabilities (Cronbach’s Alpha) for the composite scale in the current study was 0.82.

### 3.4. Self-Efficacy

The Self-Efficacy sub-scale from the Comprehensive Inventory of Thriving (CIT) and the Brief Inventory of Thriving (BIT) [29] (e.g., “I can succeed if I put my mind to it”) were used to assess the women’s self-efficacy. In addition, two items were added. One item was taken from the Global Self-Efficacy scale [102] (“I feel like I’m a valuable person, at least like other people”), and one item came from the Potency scale with a Stress Buffering Link in the Coping Stress Disease Relationship [103] (“I have little control over things that happen to me” reversed and adapted to “I have a feeling that I have influence or control over my life”). The internal consistency of the Brief Inventory of Thriving scales ranged from 0.79 to 0.87 [29]. These scales have been associated with other measures of well-being and health outcomes, demonstrating concurrent validity [29]. The internal reliability (Cronbach’s Alpha) for the scale was 0.90.

### 3.5. Meaning in Life

Two items from the Purpose and Meaning and two items from Self-Worth sub-scales from the Brief Inventory of Thriving (BIT; [29]) were used to evaluate the participants’ sense of meaning and purpose in life (e.g., “My life has a clear sense of purpose”). In addition, one item written specifically for this study was added (“The civic engagement that I am engaged in is important to other people”). The internal reliability (Cronbach’s Alpha) for the scale was 0.90.

### 3.6. Identity Exploration

Four positively worded items from the Ego Identity Process Questionnaire (EIPQ; [104]) were used to capture the women’s identity exploration in terms of value orientation, family, intimate relationships, and occupation (e.g., “I have tried to learn about different occupational fields to find the best one for me”). In addition, one item was added to assess the women’s exploration in the field of education (“I examine in-depth the field of study that suits me best”). This measure has been used with American and Dutch college students and exhibited acceptable internal reliability ranging from 0.70 to 0.90 [104,105]. The internal reliability (Cronbach’s Alpha) for the scale was 0.83.

### 3.7. Satisfaction in Life

Two items from the Satisfaction scale and two items from the Positive Emotion scale from the Brief Inventory of Thriving (BIT; [29]) were used to evaluate participants’ well-being (e.g., “I have satisfaction in my life”; “I feel happy most of the time”). The internal reliability (Cronbach’s Alpha) for the scale was 0.92.

### 3.8. Hope

The State Hope Scale [106] was used to assess the extent to which the women believed in their ability to initiate and act in different ways and achieve their goals. (e.g., “I can think of many ways to reach my current goals”). The internal reliability (Cronbach’s Alpha) for the scale was 0.72.

## 4. Results

To examine the first hypothesis, a series of Pearson correlations was conducted between the change scores for positive attitudes toward civic engagement, civic skills, political awareness, and life satisfaction and hope using the Statistical Package for the Social Sciences (SPSS) version 27. As shown in Table 1, positive medium to high correlations were found between positive attitudes toward civic engagement, civic skills, and political awareness and life satisfaction and hope. A similar picture emerged for the second hypothesis in which participants’ levels of meaning in life, self-efficacy, and identity exploration correlated positively with levels of life satisfaction and hope.

To test the mediation model in the third hypothesis, Structural Equation Modeling (SEM) using MPLUS7 [107] was applied. To build the model, three observed exogenous variables (positive attitudes toward civic engagement, political awareness, and civic engagement skills) were used as the predicting variables. Two observed variables (life satisfaction and hope) were used as the predictors. Three observed endogenous variables (women’s self-efficacy, meaning in life, and women’s score of identity exploration) were used as the mechanistic variables.

To address the second research question, indirect, direct, and full effects models (i.e., including both the indirect and direct pathways) were examined. A regression model was estimated in which the women’s levels of positive attitudes toward civic engagement, civic engagement skills, and political awareness predicted women’s life satisfaction and hope through self-efficacy, meaning in life, and identity exploration. The direct paths from women’s positive attitudes toward civic engagement, civic engagement skills, and political awareness to life satisfaction and hope were estimated (for intercorrelations, see Table 1).

Overall, the model provided a good fit with the data according to the fit criteria suggested by Hu and Bentler (1999) (χ^2^/df = 3.376/2 = 1.542, *p* = 0.185, CFI = 0.997, TLI = 0.958, SRMR = 0.024, and RMSEA = 0.017) and is presented in Figure 1, which includes the standardized estimates of the parameters in the structural model.

Analyses of the standardized total indirect and directs effects and their 95% confidence intervals with regard to life satisfaction indicated significant total indirect effects between positive attitudes toward civic engagement (estimate = 0.375, *p* = 0.000, 95% CI = 0.402 to 0.456), civic engagement skills (estimate = 0.325, *p* = 0.025, 95% CI = 0.345 to 0.386), political awareness (estimate = 0.338, *p* = 0.000, 95% CI = 0.363 to 0.406), and life satisfaction via self-efficacy, meaning in life, and identity exploration. For hope, significant total indirect effects were found between positive attitudes toward civic engagement (estimate = 0.245, *p* = 0.000, 95% CI = 0.264 to 0.309), civic engagement skills (estimate = 0.217, *p* = 0.025, 95% CI = 0.236 to 0.270), political awareness (estimate = 0.236, *p* = 0.000, 95% CI = 0.252 to 0.285).

Specific indirect effects were found between positive attitudes toward civic engagement (estimate = 0.155, *p* = 0.006, 95% CI = 0.190 to 0.217), civic skills (estimate = 0.196, *p* = 0.031, 95% CI = 0.211 to 0.238), political awareness (estimate = 0.166, *p* = 0.033, 95% CI = 0.185 to 0.217), and life satisfaction via self-efficacy. Specific indirect effects were found between positive attitudes toward civic engagement (estimate = 0.189, *p* = 0.001, 95% CI = 0.209 to 0.246), political awareness (estimate = 0.181, *p* = 0.000, 95% CI = 0.197 to 0.234), and life satisfaction via meaning in life.

Specific indirect effects were found between positive attitudes toward civic engagement (estimate = 0.086, *p* = 0.014, 95% CI = 0.209 to 0.246), civic skills (estimate = 0.131, *p* = 0.045, 95% CI = 0.146 to 0.174), political awareness (estimate = 0.112, *p* = 0.043, 95% CI = 0.126 to 0.152), and hope via self-efficacy.

Significant direct effects were found between political awareness and hope (estimate = 0.316, *p* = 0.000, 95% CI = 0.343 to 0.386). Contrary to expectations, a negative direct effect was found between positive attitudes toward civic engagement and life satisfaction (estimate = −0.107, *p* = 0.000, 95% CI = −0.090 to −0.055).

Standardized estimations of the model showed that women’s positive attitudes toward civic engagement (*β* = 0.274, *p* = 0.002), civic skills (*β* = 0.197, *p* = 0.019), and political awareness (*β* = 0.202, *p* = 0.021) predicted an increase in women’s self-efficacy, which consequently predicted an increase in women’s life satisfaction (*β* = 0.447, *p* = 0.000) and hope (*β* = 0.315, *p* = 0.000). In addition, women’s positive attitudes toward civic engagement (*β* = 0.324, *p* = 0.000), civic skills (*β* = 0.240, *p* = 0.003), and political awareness (*β* = 0.370, *p* = 0.000) predicted an increase in women’s meaning in life, which consequently predicted an increase in women’s life satisfaction (*β* = 0.454). However, although women’s civic skills (*β* = 0.240, *p* = 0.003) and political awareness (*β* = 0.318, *p* = 0.000) predicted higher levels of identity exploration, identity exploration did not predict either the level of life satisfaction or hope. Women’s level of political awareness predicted an increased level of hope (*β* = 0.288, *p* = 0.000). Nevertheless, contrary to expectations, attitudes toward civic engagement predicted a decrease in women’s life satisfaction (*β* = −0.255, *p* = 0.000) (see Figure 1).

## 5. Discussion

The current study investigated how civic engagement is associated with well-being and hope among socially excluded young women, using various self-constructs as potential mediators. This study innovates by exploring civic engagement as a strategy for broadening the well-being of young adults and disadvantaged populations, where civic engagement is rare and less likely to be initiated by youngsters themselves [108]. Furthermore, there is scant literature on ways to foster civic engagement in socially excluded young women—a largely understudied group [53].

Using cross-sectional data, the current findings indicated that self-efficacy and meaning in life play a central role in shaping women’s well-being by mediating the associations between women’s positive attitudes toward civic engagement and civic skills and their life satisfaction and hope. The intersection between the knowledge these young women gained during the program and the practical experience of the active engagement [108,109] appeared to have promoted their exploration of personal values, interests, future goals, and plans, which helped integrate the active engagement into their well-being [62,110]. These findings are aligned with previous studies that have pointed to the associations between meaning and significance in one’s life, lower levels of distress, and greater life satisfaction [56].

In addition, the findings also revealed a direct link between political awareness and hope. Political awareness is characterized by revealing and integrating issues of power, social oppression, self, collective identity, and agency. Encouraging young people to voice their intersectional experiences [111,112,113] and to actively address social concerns from the perspective of agents rather than victims [114,115,116] may restore their sense of agency, control, and hope [36,117,118].

Interestingly, although civic engagement accelerated women’s identity exploration, the drive to achieve a more vigorous sense of identity did not translate into women’s well-being or hope. The search for more consolidated self-knowledge may take longer to solidify. Alternatively, the process of identity exploration by itself does not relate directly to satisfaction in life and hope [113]. Studies have indicated that the search for identity during emerging adulthood is fraught with confusion, unease, concerns, and internal struggles [68]. This may be especially true for socially excluded populations since civic engagement may provide them with future opportunities and constraints [16,119]. However, although this encourages positive relationships, there was a negative association between positive attitudes toward civic engagement and well-being. Socially excluded women who are engaged in concerted efforts to improve their communities and society may often become disillusioned if they cannot see the fruits of their labor or if they are taught about their disadvantages without sufficient tools for generating change [120]. Frequently, sociopolitical engagement demands significant emotional time and energy, which can be associated with frustration and overwhelming experiences [36] that can hamper well-being.

### 5.1. Implications

These results have several implications. First and foremost, the findings suggest that policymakers, health care professionals, and social workers should allocate budgets and human resources to implement structured civic-engagement incubator programs to promote wellbeing and hope among disadvantaged young adult women.

Since civic engagement is a complex process that demands long-term commitment, enduring motivation, and specific knowledge and skills, practitioners should provide pre- and ongoing training dealing with the socio-political context of women and aspects of social justice. By directly addressing the systemic deprivation and power dynamics leading to marginalization, young adult women can combine their personal goals with a broader societal context, which may help promote identity exploration and a sense of meaning; thus, improving well-being. To ensure a positive experience from the engagement, the programs must be built up gradually, by encouraging these women to take small steps in practicing civic engagement skills and acquiring skills before aiming at broader efforts at social change. Clearly, organizations should refrain from blaming young women for their situation or placing the responsibility for freeing women from oppression on the young women themselves. Thus, organizations must also help these women to secure quality housing, employment, and education.

### 5.2. Limitations

Several limitations of the current study should be acknowledged. The predictive model was based on the literature on civic engagement and well-being, but the relationships from this cross-sectional design were correlational rather than causal. It is thus possible that greater well-being could contribute to greater meaning in life and self-efficacy, which in turn might promote higher levels of engagement. Longitudinal studies could help verify some of these preliminary findings. Future research could include a follow-up assessment to identify the long-term impact of the engagement more fully. Second, the results reflect the responses of socially excluded young women in Israel. Replications of the present study with diverse samples are needed before the results can be generalized to other cultures. Finally, the present study was limited to self-report measures that were often adapted to this population. Future studies would benefit from the inclusion of multiple reporters to eliminate possible self-report biases and should use the full validated measures.

## 6. Conclusions

Our model points to the benefits of civic engagement in encouraging thriving among underprivileged populations during emerging adulthood, rather than orienting efforts toward the civic engagement of young adults from middle and upper socioeconomic strata to tackle hardship and difficult life circumstances in the disadvantaged.

## Figures and Tables

**Table 1:** Correlations between variables.

		1	2	3	4	5	6	7
1	Positive Attitude toward Civic Engagement	1.00						
2	Civic Skills	0.45 ***	1.00					
3	Political Awareness	0.54 ***	0.47 ***	1.00				
4	Self-Efficacy	0.41 ***	0.42 ***	0.39 ***	1.00			
5	Meaning in Life	0.48 **	0.42 ***	0.50 ***	0.70 ***	1.00		
6	Identity Exploration	0.43 ***	0.40 ***	0.45 ***	0.44 **	0.53 ***	1.00	
7	Satisfaction in Life	0.27 ***	0.36 ***	0.39 ***	0.76 ***	0.74 ***	0.74 ***	1.00
8	Hope	0.47 ***	0.47 ***	0.55 ***	0.65 ***	0.69 ***	0.48 ***	0.52 ***

Note: *N* = 127, ** *p* < 0.01, *** *p* < 0.001.

## Data Availability

The data can be obtained upon request from the corresponding author.
